# Supplementary material for: Are carbon emissions trading and green financial instruments synergistic? -Comprehensive quantitative research based on content analysis
Source: PLoS One. 2024 Mar 7;19(3):e0298601. doi: 10.1371/journal.pone.0298601 (PMC10919601; doi:10.1371/journal.pone.0298601)
Supplement: S1 Data — (ZIP) [file pone.0298601.s001.zip › Data/地级市Z_F工作报告.zip!/─┌├╔╣┼/░═╤σ─╫╢√╩╨/└┤╫╘╣1⁄2╓┌║┼í╛┬φ┐╦╩2╛▌═°í┐_2014.docx]

各位代表：

　　现在，我代表市人民政府向大会报告工作，请予审议，并请市政协委员和列席会议的同志提出意见。

　　一、2013年工作回顾

　　过去一年，在市委的正确领导下，我们深入贯彻党的十八大精神和自治区“8337”发展思路，以“总干”精神为引领，攻坚克难，激情创业，干成了一批干部群众多年所想所盼的大事、实事，经济社会发展亮点纷呈，成效显著，实现了本届政府工作的良好开局。

　　㈠经济发展稳中向好。面对经济下行压力加大的严峻形势，把稳增长放在首要位置，着力抓项目、扩投资、兴产业，促进经济企稳回升。预计地区生产总值完成852亿元，增长9%；固定资产投资完成803.2亿元，增长14.6%；地方财政总收入完成97.6亿元，增长8%；社会消费品零售总额完成190亿元，增长12%；城镇居民人均可支配收入达到20301元，增长10%；农牧民人均纯收入达到12110元，增长13%。农作物播种面积突破千万亩，粮食总产预计达60亿斤，再创历史新高。新增设施农业2.6万亩、规模化养殖场345个。牧业年度羊饲养量达到1897万只，出栏1005万只，居全区首位。预计规模以上工业增加值完成270亿元，增长12%。神华和包钢煤焦化、联邦制药四期及五期、太平矿业二期等项目建成投产，黑猫煤化工、宇乐化工、盾安光伏二期等项目加快推进。纳林湖成为国家4A级旅游景区，总干渠20公里黄金水道投入运营，黄河旅游精彩破题。发行城投债券18亿元，东矿公司在A股上市，金融机构贷款余额达到660亿元。B型保税物流园区公路港建成，机场客运量达19万人次。县域经济、非公经济快速发展，企业总数达到11990户、个体工商户71513个。口岸过货量1120万吨，其中铜精粉10万吨。引进国内到位资金346亿元，利用外资7175万美元。

　　㈡城乡面貌明显变化。采取政府、企业、社会多元投入方式，实施了一批打基础、利长远的重点工程。中心城区和县城人居环境大幅改善。25公里新华街综合改造全线完工，曙光街、河套大街西段部分建成通车。双河新区建设稳步推进，金川大桥、金川南路和黄河湿地公园一期工程建成投用。全市59条街路和18个出口改造基本完成。城市绿化、美化、亮化水平显著提高，干净、整洁成为城市常态。改造重点集镇19个，建设新农村新牧区示范点101个，完成村庄整治337个，镇容村貌大为改观。4条公路纳入国家公路网规划，新改建公路1020公里，临河绕城、五原至刘召一级公路竣工通车，临河客运新站投入运营。乌力吉风电汇集站、金泉220千伏等输变电工程建成投用。50万亩中低产田改造顺利实施，100万亩农村土地整治全面完成。三年排水改造首战告捷，整治沟道4272公里。

　　㈢水、绿、文化彰显特色。临河镜湖、杭后润昇湖、五原隆兴湖、磴口奈伦湖、前旗东河水系、中旗滨河水系、后旗迎宾湖等一批湿地保护与恢复工程基本完工，新增湿地10万亩。完成林业生态建设77.3万亩，实施了京藏高速、黄河堤防公路、临陕路、212省道等一批精品绿化项目，造林投入、面积和质量均创历史最好水平。乌梁素海水质明显改善，乌拉特草原阶段性禁牧和草畜平衡面积达到7258万亩，乌兰布和沙漠生态治理17万亩。新建改造了一批彰显文化特色的街路和建筑，黄河水利文化博物馆、蒙古部落文化园区、临河石艺街、后旗那达慕体育场等项目竣工投用。成功举办沿黄、环镜湖公路自行车赛、内蒙古首届中西部民歌大赛，《月照金河套》荣获第十届内蒙古草原文化节优秀剧目奖，临河区被评为自治区文明城区，乌拉特后旗获自治区文明旗县城提名资格。通过探索和努力，我们在特色发展上找到了突破口，水、绿、文化正在成为巴彦淖尔的靓丽名片。

　　㈣民生和社会事业全面进步。在财力困难的情况下，优先保障民生支出，共计达到134.8亿元，占财政总支出的70%。新增城镇就业1.3万人，农村牧区劳动力转移就业16.5万人。基本养老、医疗保险参保人数分别达到103万人、160万人。城乡低保、五保供养、“三无”人员保障标准稳步提高，为低收入农牧户发放煤补1.36亿元。企业退休人员养老金月人均增加155元，发放了机关事业单位工作人员带薪休假和通讯补贴。下大力气解决供水、供热、供气等方面的问题，新建改造自来水厂5个、供水管网140公里，新增集中供热面积200万平方米、天然气入户2.1万户。开工建设保障性安居工程1.55万套。投入扶贫资金4.1亿元，3.3万贫困人口稳定脱贫。新建和改扩建幼儿园31所，河套学院新校区和市医院新址开工建设，蒙医院投入运营，河套农牧业技术研究院挂牌成立。实施了13个苏木乡镇基础设施项目，新建7个新型社区服务中心。人口计生、妇女儿童、老龄、残疾人等事业健康发展。社会治理、平安创建成效显著，治安环境明显好转。一批信访积案得到化解，安全生产形势稳定，食品药品监管有力。民族宗教、双拥共建等均取得新成绩。

　　㈤政府自身建设得到加强。认真落实改进作风的各项规定，深入开展正风肃纪活动，严格接待标准，精减会议文件，反对铺张浪费，以政风的好转促进了民风和社会风气的转变。公务用车治理和办公用房整顿落实到位，“三公”经费支出减少41％。主动接受人大依法监督和政协民主监督，共办理人大代表建议104件，政协委员提案257件，办结率均达到100％。机构改革不断深化，15个国营农牧场移交旗县区管理，文化、广电、新闻出版“三局合一”改革到位。大力简政放权，取消下放行政审批事项102项，市公共资源交易平台启动运行。审计、监察工作力度加大，反腐倡廉建设深入推进。

　　各位代表，过去一年，是我们咬定目标不放松、凝心聚力干事业的一年；是全市上下负重前行、奋勇争先，各项工作取得新突破的一年；是巴彦淖尔影响力和知名度得到提升，各族人民自豪感和凝聚力不断增强的一年。这些成绩的取得，是自治区党委、政府和市委正确领导的结果，是市几大班子和衷共济、团结奋进的结果，是全市人民齐心协力、顽强拼搏的结果。在此，我代表市人民政府，向所有为巴彦淖尔建设和发展作出贡献的同志们、朋友们，表示衷心的感谢和崇高的敬意！

　　过去一年，政府工作还有许多不尽如人意的地方。比如，在经济下行等因素影响下，我们不得不对一些主要经济指标进行了调整；有的行业和企业生产经营困难，短期内走出困境的难度还比较大；新开工项目和大项目少，财政增收乏力，政府性债务压力比较大；特色产业规模小、层次低，优势还没有充分发挥出来；设施农业利用率不高，任务完成得不够理想；关系群众切身利益的就业、住房、教育、医疗等方面还存在不少问题，征地拆迁、利益纠纷等社会矛盾还比较突出；行政审批多、审批慢、部分机关和工作人员办事效率不高的问题仍然存在，等等。新的一年，我们一定加倍努力，寻求突破，在真抓实干中再创佳绩。

　　二、2014年主要工作

　　今年是深入贯彻党的十八届三中全会精神的开局之年，也是实现“十二五”奋斗目标的关键一年。中央和自治区新一轮改革的全面实施，必将为科学发展注入强大的动力和活力。中央经济工作、城镇化和农村工作会议，为我们加快推进新型工业化、新型城镇化和农牧业现代化指明了方向。国家和自治区对生态环保、“三农三牧”的空前重视，为我们发挥优势、走绿色发展之路带来了千载难逢的机遇。自治区“8337”发展思路的进一步深化和完善，使我们更加明确了发展定位和方向，加之多年来，巴彦淖尔发展成果的积累，我们已经站在了新的起点上。只要我们坚持不懈，凝神静气、心无旁骛做好自己的事，就一定能够发挥后发优势，实现赶超崛起。

　　今年政府工作的总体要求是：以科学发展观为指导，认真贯彻党的十八届三中全会、自治区党委九届九次、十次全委会、市委三届八次全委会暨经济工作会议精神，全面落实自治区“8337”发展思路，大力弘扬“总干”精神，以深化改革开放为动力，以绿色发展为主攻方向，以投资和项目建设为主要抓手，大力发展县域经济和非公经济，坚持做好“水、绿、文化”三篇文章，积极创新社会治理，努力增加人民福祉，建设富裕、美丽、幸福、文明、活力巴彦淖尔。

　　主要预期目标是：全市生产总值增长9.5%，固定资产投资增长16%，社会消费品零售总额增长12 %，公共财政预算收入增长7%，城镇居民人均可支配收入和农牧民人均纯收入分别增长10 %和13 %。城镇登记失业率控制在4％以内。居民消费价格涨幅控制在3.5%左右。单位生产总值能耗和二氧化碳排放量分别下降2%和3%。

　　确定上述目标，基本着眼点是积极稳妥、稳中求进、改革创新，总体上达到或高于自治区平均水平。中央强调“不以GDP论英雄”，并不是不要GDP，而是不唯GDP，自治区也确定了保持经济合理增速的总基调。我市是欠发达地区，发展不足仍然是当前的主要矛盾，在加快发展上决不能有丝毫动摇，必须保持一定的增长速度，才能解决好财政增收、民生改善等问题，但发展必须是有质量、有效益、可持续的发展，一句话就是“绿色发展”。

　　根据总体要求和预期目标，要紧紧围绕改革创新、经济发展、社会和谐三大主题，着力深化五项改革，抓好六个重点，提升七个方面的工作水平。

　　深化五项改革：改革是最大的红利。要把改革创新贯穿于经济社会发展的各个领域各个环节，积极稳妥、量力而行推进各项改革，努力使经济社会发展更有效率、更加公平、更可持续。

　　一是经济体制改革方面，要处理好政府与市场的关系，使市场在资源配置中起决定性作用。全面落实促进非公经济发展的政策措施。推进工商登记制度改革，放宽市场主体准入条件。坚持农村牧区基本经营制度，鼓励发展多种形式的规模经营。完成农村牧区土地确权登记颁证工作，探索建立土地承包经营权抵押担保制度。深化财税体制改革，建立政府债务管理及风险预警机制，严格控制新增债务。争取中旗、后旗、磴口农村信用社改制为农商行。深化户籍制度改革，稳步推进农牧业转移人口市民化。

　　二是行政体制改革方面，要加快政府职能转变，把工作重心转移到公共服务、市场监管、社会管理、环境保护上来。推进行政审批制度改革，承接好自治区下放的审批事项，取消和下放一批审批事项，把该放的权力放到位，该管的事务管住管好，防止出现“一放就乱，一管就死”的现象。按照行政监管与交易业务相分离的原则，完善公共资源交易平台建设。整合卫生、人口和计生部门机构职责，理顺食品药品监管机制。严格控制机构编制，推进事业单位分类改革，扩大政府购买服务范围。做好国有农牧场管理体制改革后续工作。

　　三是文化体制改革方面，要理顺文化管理体制，抓好文化市场综合执法机构改革。建立现代文化市场体系，支持各种形式小微文化企业发展。推动公共图书馆、博物馆、科技馆等公益性文化事业单位改革，促进基本公共文化服务标准化、均等化。深化广电、报业传媒改革，推进文化发展投资市场化进程。

　　四是社会体制改革方面，要大力促进教育公平，理顺义务教育管理体制，努力缩小义务教育阶段城乡、校际差距。完善城镇优质学校对口帮扶农村牧区学校和薄弱学校制度，推进县域内校长教师交流轮岗，提高农村牧区教师待遇。不设重点学校重点班，逐步缩小班容量，标本兼治减轻学生课业负担。整合职业教育、就业培训等资源，促进人才、资金优化配置。完善社会保险制度，将有条件参保的各类人员尽可能纳入保险范围。深化医药卫生体制改革，扩大基本药物制度实施范围。适时实施一方是独生子女的夫妇可生育两个孩子的政策。改进社会治理方式，以网格化管理、社会化服务为方向，完善基层综合服务管理平台。严格落实重大项目社会稳定风险评估机制，健全及时就地解决群众合理诉求机制，依法规范信访秩序。

　　五是生态文明体制改革方面，要坚定不移实施主体功能区制度，划定生态红线，建立水系、湖泊、森林、荒地等自然生态空间开发保护制度。完善草原生态保护补助奖励长效机制，巩固和扩大集体林权制度改革成果。科学配置生产、生活、生态用水，严控超采地下水。完善污染物排放许可制，对造成生态环境损害的责任者严格实行赔偿制度，依法追究刑事责任。

　　抓好六个重点：就是牢牢扭住经济建设这个中心不动摇，按照“稳中有进、稳中向好”的要求，抓好经济发展方面的六项重点工作。

　　一是以项目建设为抓手，促进工业经济扩量提质。牢固树立抓项目就是抓发展的理念，围绕建设“五个基地”，做大优势产业规模，加快发展县域经济。进一步优化产业布局：沿山旗县工业园区集中发展冶金、化工、电力等产业；套区发展农畜产品加工、高新技术等无污染产业。专项推进重大项目建设，完成工业固定资产投资400亿元以上，规模以上工业增加值增长13％。开工建设神华煤焦化二期、中旗20万吨铜冶炼、大中200万吨铁精粉采选等项目，加快实施黑猫煤化工、宇乐化工、百万千瓦风电基地等项目，确保盾安太阳能电池组件、海明矿业铁精粉采选、蒙羊肉羊产业园等项目建成投产。积极推进河套酒业技改扩建、神东2×35万千瓦低热值煤发电、中金集团黄金精炼、浙能60万吨甲醇、华拓矿业600万吨铁精粉等项目建设。开工或建成黑柳子500千伏、临河西220千伏等14项输变电工程。大力发展清洁能源产业，新增风电装机80万千瓦、光伏发电20万千瓦。配套完善园区供水、供电、污水处理等基础设施，打造一批花园式企业和园区。认真落实扶持企业发展的政策措施，强化企业运行调度，帮助停产半停产企业恢复生产。引导企业加大技术改造力度，促进产业产品升级。加快非公经济和小微企业发展，设立小微企业发展专项资金，建成杭后、五原小微企业创业园。

　　二是以绿色有机为方向，加快发展现代农牧业。坚持打生态牌，走特色路，当好自治区绿色农畜产品生产加工输出基地排头兵，积极向国家级绿色有机食品生产基地迈进。提高粮食综合生产能力，粮食总产稳定在60亿斤以上。大力发展绿色有机种植，“三品”标准化基地达到360万亩以上。实施130万亩高标准基本农田建设项目，完成灌区排沟整治3143公里、节水改造50万亩，做好节水抗旱工作。建设标准化科技示范园区10个，培训农牧民科技人员50万人次。坚定不移推进设施农业建设，在条件成熟的地区集中连片布局，建设临河八一、五原隆兴昌、杭后春光3个万亩设施农业示范基地，提高设施农业利用率和收益率。建设规模化养殖场350个，建成富川、草原宏宝、力农、京原4个年出栏10万只以上的肉羊养殖园区。争取实施国家新增出栏1000万只优质肉羊生产基地项目。全市羊饲养量达到2200万只，出栏1200万只。新增有机奶牛1万头，有机奶产量达到全国第一。提高农牧业产业化水平，强化“龙头＋基地＋品牌”联结机制，集中打造“河套”、“巴盟”优势特色品牌，申报乌拉特山羊、河套小麦、华莱士等6个国家地理标志产品登记保护，争取更多产品进入高端市场。千方百计提高农牧业经营效益，拓展农牧民增收渠道，让农牧业成为有奔头的产业，让农牧民成为体面的职业。全力抓好农畜产品质量安全，建成市农畜产品综合检验检测中心，加快建设农畜产品质量和食品安全追溯体系，确保广大人民群众“舌尖上的安全”。

　　三是以特色旅游为突破，提升第三产业发展水平。依托黄河、湿地等生态资源优势，打造黄河旅游文化集中展示区和体验区。完善黄河湿地旅游景区设施，建好走西口•民俗文化村，力争建成国家5A级景区。配套完善纳林湖旅游设施，湖区面积逐步扩大到5万亩。启动实施杭后双庙沙漠旅游项目，加快大桦背、乌梁素海、牧羊海等旅游资源开发，发展白彦花等美丽村镇特色游。开通季节性旅游航班，拓展旅游客源市场，全年接待游客300万人次以上，旅游总收入突破30亿元。开工建设临哈高速、甘其毛都至海流图一级公路、中旗通勤机场等项目，完成青山至乌根高勒、海流图至五原一级公路续建工程。实施四季青农畜产品物流交易中心项目，建成羊畜产品交易平台。加快建设红星美凯龙、维多利等城市商业综合体。支持和引导金融机构扩大融资规模，鼓励引进区内外银行等金融机构在我市设立分支机构。推进文体、信息、健康等服务业发展，培育新兴消费业态。开展第三次全国经济普查工作。

　　四是以口岸经济为依托，构建对外开放新格局。抓好对蒙贸易、入境资源加工和口岸新城建设，打造向北开放桥头堡和重要经济增长极。完善通关查验、仓储物流设施，建成铁路、公路综合监管区。建立定期会晤机制，加强与蒙方沟通协作，扩大口岸过货量和贸易额，推进煤、铜资源落地加工。配套完善口岸城镇基础设施，提升综合服务能力。完成互市贸易区一期工程，繁荣口岸小额贸易。大力改善投融资环境，加大招商引资力度。抓住自治区深化港、澳、台交流合作的契机，积极承接东南沿海产业转移，跟踪落实签约和重点合作项目。严把项目准入关，决不引进污染企业。加强与呼包鄂银等周边地区的经济协作，在资源、技术、资金和市场等方面争取更大份额。支持乳、肉、绒、番茄、脱水菜等农畜产品拓展国际市场，争取籽仁基地成为国家外贸转型升级示范基地。进出口总额达到14.5亿美元，增长8%。

　　五是以新型城镇化为引领，统筹抓好城乡建设。按照生产空间集约高效、生活空间宜居适度、生态空间山清水秀的总体要求，协调推进中心城区、县城、集镇和新农村新牧区建设。继续坚持市区共建，加大中心城区建设力度，打通金川北路、河套大街胜利路以西剩余段落。本着尊重自然、顺应自然、天人合一的理念，依托现有湿地、田园等自然风光，规划建设好双河新区，让城市融入大自然。启动先锋新桥、先锋南路、敕勒大街、河套学院新校区主体工程建设。探索以群众为主体、政府为主导的居民“自治改造”新模式，完成棚户区改造8730户。专项推进棚户区改造尾留工程，做好居民回迁安置工作。实施乌拉山东、海流图南、隆兴昌西等14个城镇出入口综合整治。全市新增集中供热面积150万平方米、城市绿化面积1200亩、天然气入户1.5万户，完成公厕旱改水30座，实现垃圾无害化处理全覆盖。抓好19个集镇和100个新农村新牧区示范点建设。要注重保留原始风貌，多栽树、少拆房、不填湖，尽可能在原有村庄形态上改善居民生活条件，让居民望得见山、看得见水、记得住乡愁，让农村牧区真正成为美丽乡村、幸福家园。深入开展“城市建设管理年”活动。严肃规划执法，依法节约集约用地。加强市政公共设施维护管理，强化交通综合治理，启动公交改革。整顿规范物业管理市场，做好住宅小区电梯维修、保洁保安等工作。大力开展城乡环境卫生综合整治，加大“四堆”（垃圾堆、柴草堆、土堆、粪堆）清理力度，让群众生活得更干净、更整洁、更舒适。

　　六是以天蓝地绿水清土净为目标，加强生态建设和环境保护。围绕建设祖国北疆重要生态安全屏障的目标，依托天保工程、退耕还林、京津风沙源治理等项目，大规模开展植树造林，高标准实施通道、城镇、村屯等重点区域绿化，完成林业生态建设100万亩。因地制宜发展林果业，建设种苗基地2万亩。治理乌兰布和沙漠17万亩，大力发展沙产业，新增肉苁蓉、有机酿酒葡萄基地2万亩。坚持政策引导、群众自愿、宜移则移原则，运用新型城镇化理念，科学做好生态移民工作。加强湿地、湖泊的保护与利用，细化管理，适时补水，积极发展水产养殖业。大力实施乌梁素海网格水道、分洪补水等综合治理工程。提高资源综合利用水平，建设一批循环经济示范园区和企业。落实节能减排任务，抓好矿山冶金、电力等重点行业节能降耗。加快临河第二污水处理厂建设，实现全市污水处理厂稳定运营、达标排放。大力倡导绿色出行、低碳生活，使节能、环保深入人心，让保护好河套这方净土成为每个人的责任。

　　提升七个方面的工作水平：就是把民生作为一切工作的出发点和落脚点，努力做好七个方面的工作。

　　一是提升社会保障水平。全市安排社会保障资金5.1亿元，较上年增加6631万元。城镇职工、居民医保政策内报销比例分别达到85%、70%以上；新农合人均筹资标准提高到420元。城市低保标准月人均达到441元，农村牧区低保标准年人均达到3265元；五保对象集中和分散供养标准年人均分别提高900元、500元；城镇“三无人员”集中和分散供养标准月人均分别提高130元、120元；孤儿集中和分散供养标准月人均分别提高190元、150元。加快城乡养老服务业发展，新建2所市级老年人养护院、15处农村牧区互助幸福院。实施百姓安居工程，开工各类保障性住房4615套，完成农村牧区危旧房改造1.5万户。打好扶贫开发攻坚战，认真落实规划、项目、干部“三到村三到户”帮扶举措，安排扶贫专项资金3600万元以上，帮扶3万贫困人口脱贫。

　　二是提升创业就业水平。大力实施创业就业工程，多渠道开发就业岗位，新增城镇就业1.2万人，农村牧区劳动力转移就业16万人。发放小额担保贷款2亿元，储备各类人才760名，完成城乡职业技能培训1.6万人。健全工资正常增长机制，多渠道增加职工收入。严格执行最低工资标准和工资集体协商制度，维护职工合法权益。

　　三是提升教育发展水平。 始终坚持教育优先发展，努力办好人民群众满意的教育。全市安排教育专项4.15亿元，较上年增加1.01亿元。完成学前教育三年行动计划，改扩建中心集镇公办幼儿园10所。推进义务教育均衡发展，提升高中教育质量，加快特殊教育学校标准化建设，启动建设市直民族教育园区。多层次资助困难家庭学生，让每一个孩子都有学上、上好学。

　　四是提升医疗卫生水平。医疗卫生关系群众幸福安康，要努力使群众享受优质的医疗服务。全市安排医疗卫生专项5.4亿元，较上年增加4997万元。加快市医院新址、精神病院和旗级中蒙医院建设，完成6所苏木镇卫生院、40所嘎查村卫生室建设。实施中蒙医预防保健项目和“健康保障小药箱”进牧户工程。建成一体化管理卫生信息平台，启动家庭医生服务模式，实现市域内医疗资源共享。

　　五是提升文化体育发展水平。加快实施文化服务惠民工程，全市安排文化体育专项2.01亿元，较上年增加8652万元。启动建设市体育馆和河套文化产业园区，市广电传媒中心入驻使用，新建和完善基层文化活动室130个。继续办好沿黄、环镜湖公路自行车赛、河套文化艺术节，做好阴山岩刻申遗工作。深入开展精神文明创建活动，培育和践行社会主义核心价值观，提高公民文明素质。

　　六是提升科技服务水平。全市安排科技专项2282万元，较上年增加1030万元。启动1家产业技术创新战略联盟，新增2家自治区级企业研发中心。加强知识产权保护，完成专利授权200件以上。加快建设产学研相结合的科技创新体系，推进科技成果转化和推广。大力实施人才强市工程，重视解决优秀人才流失问题，统筹抓好各类人才队伍建设。

　　七是提升社会治理水平。深入推行“三一四全”工作法和村民代表会议常设制，有效预防和化解社会矛盾。全面实施平安创建工程，完善社会治安防控体系，严厉打击各类违法犯罪活动。加强派出所基础建设，建成“护城河工程”工作站9个。完善应急管理体系，妥善应对网络舆情。强化安全生产监管，坚决遏制重特大事故发生。全面落实党的民族宗教政策，大力支持国防和军队建设，筑牢祖国北疆安全稳定屏障。

　　各位代表，打铁还需自身硬。我们要进一步加强法治政府、服务政府、廉洁政府建设，把党和人民赋予的神圣职责履行好。全面推进依法行政，自觉接受人大及其常委会的法律监督、政协的民主监督，认真办理人大代表建议和政协委员提案，广泛听取民主党派、工商联、无党派人士和老干部的意见。深入开展党的群众路线教育实践活动，集中整治形式主义、官僚主义、享乐主义、奢靡之风。进一步提升行政服务水平，切实解决有令不行、有禁不止和慢作为、不作为、乱作为的问题。严格落实中央、自治区和市委改进作风的各项规定，进一步转变文风、会风、作风。认真执行国务院“约法三章”，厉行节约，反对浪费，严禁新建政府性楼堂馆所，财政供养人员只减不增，公务经费在上年基础上压缩5%。全面落实党风廉政建设责任制，强化权力运行的监督制约，把权力关进制度的“笼子”，以为民务实清廉的形象取信于民。

　　各位代表，巴彦淖尔正处于绿色发展的机遇期、不进则退的攻坚期，唯有奋力前行、敢于担当，方能不辱使命、创造辉煌。让我们在市委的坚强领导下，大力弘扬“总干”精神，团结带领全市各族人民，紧跟时代步伐，锐意进取，开拓创新，谱写巴彦淖尔改革开放和现代化建设的新篇章！
